# Supplementary material for: Phytoextracts for Human Health from Raw and Roasted Hazelnuts and from Hazelnut Skin and Oil: A Narrative Review
Source: Nutrients. 2023 May 23;15(11):2421. doi: 10.3390/nu15112421 (PMC10255299; doi:10.3390/nu15112421)
Supplement: Supplementary file 1 [file nutrients-15-02421-s001.zip › nutrients-2381821-supplementary.pdf]

Table S1

Analysis of aminoacids, proteins, sugars, fiber, carbohydrates and fats quantities contained in the 1) raw hazelnut with skin (R_S), and 2) toasted hazelnut without skin (T_WS) from Turkish grown in Turkey (Tombul).

A

**Turkish hazelnut grown in Turkey**			
**Tombul variety**	**Raw with Skin (R_S)**	**Toasted Without Skin (T_WS)**	**Text pages**
**ESSENTIAL AMINO ACIDS**	**g/100g**	**g/100g**	
Isoleucine	0.58 (1)	nr	#4,6-7
**Leucine**	**1.07**(1)	nr	#4,6-7
Lysine	0.41 (1)	nr	#4,6-7
Methionine	0.23 (1)	nr	#4,6-7
Phenylalanine	0.66 (1)	nr	#4,6-7
Threonine	0.53 (1)	nr	#4,6-7
Tryptophan	0.04 (1)	nr	#4,6-7
Valine Average/ds/cv	0.71 (1) 40.60 (2)	34.00 (3)-32.60 (4) /0.99/0.03	#7
**Total Essential amino acids (g/100g)**	**4.23** (1)	nr	

B

**Turkish hazelnut grown in Turkey**			
**Tombul variety**	**Raw with Skin (R_S)**	**Toasted Without Skin (T_WS)**	**Text pages**
**NON-ESSENTIAL AMINO ACIDS**	**g/100g**	**g/100g**	
Alanine	0.70 (1)	nr	#4,7
**Arginine**	**2.16** (1)	nr	#4,7
Asparagine	nr	nr	nr
**Aspartic acid**	**1.52**(1)	nr	#4,7
Cysteine	0.46 (1)	nr	#4,7
**Glutamic acid**	**3.13** (1)	nr	#4,7
Glutamine	nr	nr	nr
Glycine	0.71 (1)	nr	#4,7
Histidine	0.45 (1)	nr	#4,7

Proline	0.56 (1)	nr	#4,7
Serine	0.65 (1)	nr	#4,7
Tyrosine	0.53 (1)	nr	#4,7
**<u>Total Non-essential amino acids (g/100g)</u>**	**<u>10.87</u>**	nr	#7

C

**<u>Total amino acids (g/100g)</u>**	**<u>15.10</u>**	nr	#7

D

Turkish hazelnut grown in Turkey							
**Tombul –Viba Sweet varieties**	**Raw with Skin (R_S)**			**Toasted Without Skin (T_WS)**			**Text pages**
**<u>PROTEINS</u>**	**relative %**			**relative %**			
Protein amount (%)	0.47 (2)/ 14.2 (7)/ 16.60 (10)/			17.8 (5)/15.4 (8)/ 15.20 (9)/ /0.55 (3)/0.58 (4)			**#7**
**<u>Protein amount</u>**	**av.% <u>10.42</u>**	SD 8.7	CV 0.8	**av.% <u>9.9</u>**	SD 8.6	CV 0.8	**#7**
	**g/100g**			**g/100g**			
Total Proteins	**15.35** (1) / **17.42** (6)			nr			**#7**
**<u>Total Proteins</u>**	**av. 16.39**	s.d. 1.46	CV 0.09	nr			**#7**

E

Turkish hazelnut grown in Turkey			
**Tombul variety**	**Raw with Skin (R_S)**	**Toasted Without Skin (T_WS)**	**Text pages**
**<u>SUGARS</u>**	**g/100g**	**g/100g**	
**Glucose (monosaccharide)**	**0.11** (1)	nr	**#7**
**Fructose (monosaccharide)**	**0.14** (1)	nr	**#7**
**Myo-inositole (monosaccharide)**	**0.04** (1)	nr	**#7**
**Sucrose (disaccharide)**	**2.67** (1)	nr	**#7**
Raffinose (trisaccharide)	0.14 (1)	nr	**#7**
Stachyose (tetrasaccharide)	0.48 (1)	nr	**#7**
**Total sugars**	**<u>3.58</u>** (1)	nr	**#7**
Simple Sugars	2.96 (1)	nr	**#7**
Complex Sugars	0.62 (1)	nr	**#7**

F

Turkish hazelnut grown in Turkey			
**Tombul variety**	**Raw with Skin (R_S)**	**Toasted Without Skin (T_WS)**	**Text page**
**FIBER**	**g/100g**	**g/100g**	#7
Insoluble fiber	10.67 (1)	nr	#7
Soluble fiber	2.21 (1)	nr	#7
**Total fiber**	**12.88**	nr	#7
**Carbohydrates (simple/complex sugars and fiber)**	**17.30** (1)	nr	#7

G

**Tombul variety**	**Raw with Skin (R_S)**	**Toasted Without Skin (T_WS)**	**Text page**
**FATS**	**g/100g**	**g/100g**	
Total fats %	63.60(7)/ 61.90 (10)	64.6 (8)/ 64.5 (9)	#8, #9
**Total fats (g/100g)**	**62.8 (1)**	**64.1**	#8, #9

Table S1 Legend

Publications	State of production	Hazelnut type	Hazelnut processing	Skin
1) Alasalvar et al. 2003 [1]	Turkey	Tombul	**(R_S)**	**Yes**
2) Stuetz et al. 2017 [2]	Turkey	Viba Sweets	**(R_S)**	**Yes**
3) Stuetz et al. 2017 [2]	Turkey	Viba Sweets	**(T_WS) at 140 °C**	**No**
4) Stuetz et al 2017 [2]	Turkey	Viba Sweets	**(T_WS) at 170 °C**	**No**
5) Erdemir et al. 2014 [3]	Turkey	Tombul	**(T_WS)**	**No**
6) Ozenc et al.2014 [4]	Turkey	Tombul	**(R_S)**	**Yes**
7) Locatelli et al. 2015 [5]	Turkey	Tombul	**(R_S)**	**Yes**
8) Locatelli et al. 2015 [5]	Turkey	Tombul	**(T_WS) at 160 °C**	**No**
9) Locatelli et al. 2015 [5]	Turkey	Tombul	**(T_WS) at 180 °C**	**No**
10) Schlormann et al. 2015 [6]	Turkey	Tombul	**(R_S)**	**Yes**

Concentration analysis of essential, non-essential, total amino acids (A_B_C), total proteins (D), simple, complex, total sugars (E), insoluble/soluble/total fiber and total fats (F) in the *Turkish* Tombul hazelnut by comparing data of raw hazelnut with skin (R_S) v/s toasted hazelnut without skin (T_WS). Roasting temperatures and publications showing roasting times (ranging from 15 to 30 minutes) were indicated. Data were obtained from six different publications as indicated in

the table above. Nutrient concentrations showed in the tables were expressed as g/100grams of hazelnut product or %. The acronym “nr” means “value not recorded”.

Table S2

Analysis of fatty acid quantity contained in 1) raw hazelnut with skin (R_S), and 2) toasted hazelnut without skin (T_WS) from different origin: (2A) Turkish grown in Turkey (Tombul), (2B) Italian hazelnut grown in Italy (TGT-TG) and (2C) Italian hazelnut grown in Chile (TGT-Chile).

A

**FATTY ACIDS**	**Turkish Hazelnut grown in Turkey. TOMBUL**								
	**Raw with Skin (R_S)**				**Toasted Without Skin (T_WS)**				**Text page**
	**TOMBUL**	**Average**	SD	CV	**TOMBUL**	**Average**	SD	CV	
	**relative %**				**relative %**				
C14:0	0.0	/	/	/	nr	/	/	/	nr
C16:0	5.55 (7), 5.10 (10),	5.33	0.32	0.1	5.54 (8), 5.57(9)	5.56	0.32	0.10	#8
C16:1 Δ9	0.20 (10)	0.20	/	/	nr	/	/	/	#8
C18:0	2.80 (7), 2.00 (10),	2.40	0.57	0.2	2.73 (8), 2.54 (9)	2.63	0.13	0.10	#8
C18:1 Δ9	80.70 (7), 82.60 (10),	81.65	1.34	0.0	82.00 (8), 80.30 (9)	81.15	1.20	0.0	#8
C18:1 Δ11	1.39 (7)	1.39	0.12	/	1.15 (8), 1.24 (9)	1.20	0.06	0.10	#8
C18:3 n-3 (ALA)	0.10 (10)	0.10	/	/	nr	/	/	/	#8
C18:2 n-6 (LA)	9.61(7),8.70 (10)	9.15	0.2	/	8.49 (8), 10.33 (9)	9.41	1.30	0.10	
C20:0	0.10 (10)	0.10	/	/	nr	/	/	/	#8
C20:1 e11	0.10 (10)	0.10	/	/	nr	/	/	/	#8
C22:0	0.0 (10)	0.0	/	/	nr	/	/	/	#8
C24:0	0.0 (10)	0.0	/	/	nr	/	/	/	#8
**Total SFA (%)**	7.83	**7.83**	0.89	0.11	8.19	**8.19**	0.45	0.05	#8
**Total MUFA (%)**	83.34	**83.34**	1.46	0.02	82.35	**82.35**	1.26	0.015	#8
**Total PUFA (%)**	9.25	**9.25**	0.2	0.02	9.41	**9.41**	1.30	0.10	#8

B

FATTY ACIDS	Italian "TGT hazelnut" grown in Italy. TGT_TG										
	Raw with Skin (R_S)					Toasted without Skin (T_WS)					Text page
	TGT_ Piedmont	TG_ Campania	Av.	SD	CV	TGT_ Piedmont	TG_ Campania	Av.	SD	CV	
	relative %					relative %					
C16:0	6.16 (7*)	5.55 (7*)	5.86	0.43	0.07	6.21 (8*), (9*)	5.5 (8*), (9*)	5.88	0.47	0.08	#8, #9
C18:0	3.14 (7*)	2.85 (7*)	3.0	0.21	0.07	3.13 (8*), (9*)	3.03 (8*), (9*)	3.08	0.07	0.02	#8, #9
C18:1 Δ9	82.55 (7*)	80.2 (7*)	81.38	1.66	0.02	82.72 (8*), (9*)	80.35 (8*), (9*)	81.54	1.68	0.02	#8, #9
C18:1 Δ11	1.33 (7*)	1.77 (7*)	1.55	0.31	0.20	1.33 (8*), (9*)	1.13 (8*), (9*)	1.23	0.14	0.11	#8, #9
C18:2	6.86 (7*)	10.9 (7*)	8.88	2.86	0.32	7.1 (8*), (9*)	10 (8*), (9*)	8.55	2.05	0.24	#8, #9
**Total SFA (%)**	9.3	8.4	**8.85**	0.64	0.07	9.34	8.53	**8.96**	0.54	0.06	#8, #9
**Total MUFA (%)**	83.88	81.97	**82.93**	1.97	0.02	84.03	81.48	**82.77**	1.82	0.02	#8, #9
**Total PUFA (%)**	6.86	10.9	**8.88**	2.86	0.32	7.1	10	**8.55**	2.05	0.24	#8, #9

C

FATTY ACIDS	Italian "TGT hazelnut" grown in Chile. TGT_Chile									
	Raw with Skin (R_S)				Toasted without Skin (T_WS)					Text page
	TGT_Chile	Av.	SD	CV	TGT_Chile		Av.	SD	CV	
	relative %				relative %					
C16:0	6.92 (7+)	6.92	0.01	0.00	7.16 (8+)	7.15 (9+)	7.15	0.01	0.0	#8, #9
C18:0	2.98 (7+)	2.98	0.09	0.03	3.0 (8+)	3.35 (9+)	3.18	0.25	0.07	#8, #9
C18:1 Δ 9	80.8 (7+)	80.8	0.4	0.00	80.2 (8+)	80.4 (9+)	80.30	0.14	0.0	#8, #9
C18:1 Δ11	1.61 (7+)	1.61	0.06	0.04	1.65 (8+)	1.53 (9+)	1.60	0.08	0.05	#8, #9
C18:2	7.73 (7+)	7.73	0.26	0.03	7.97 (8+)	7.57 (9+)	7.78	0.28	0.03	#8, #9
**Total SFA (%)**		**9.90**	0.10	0.01			**10.33**	0.26	0.02	#8, #9
**Total MUFA (%)**		**82.4**	0.46	0.00			**81.90**	0.22	0.00	#8, #9
**Total PUFA (%)**		**7.73**	0.26	0.03			**7.78**	0.28	0.03	#8, #9

Table S2A, B, C Legend

Publications	State of production	Hazelnut type	Hazelnut processing	Skin
7) Locatelli et al. 2015 [5]	7) Turkey	Tombul	**(R_S)**	**Yes**
	7*) Italy	TGT_TG		
	7+) Chile	TGT_Chile		
8) Locatelli et al. 2015 [5]	8) Turkey	Tombul	**(T_WS) at 160 °C**	**No**
	8*) Italy	TGT_TG		
	8+) Chile	TGT_Chile		
9) Locatelli et al. 2015 [5]	9) Turkey	Tombul	**(T_WS) at 180 °C**	**No**
	9*) Italy	TGT_TG		
	9+) Chile	TGT_Chile		
10) Schlörmann et al. 2015 [6]	Turkey	Tombul and others	**(R_S)**	**Yes**

Data obtained from two different publications as above indicated. Fatty acid quantifications were expressed as relative percentage of total FAME (fatty acid methyl esters). Individual compounds were quantified as relative percentage (dividing the range for mean value and multiplying for 100, expressed as average +nr- SD, as described in Locatelli M. et al.2015 and Schlörmann et al

2015). Where necessary, concentration average together with SD and CV were calculated separately. Total saturated fatty acid (SFA), total mono-unsaturated fatty acid (MUFA) and poly-unsaturated fatty acid (PUFA) were calculated by the sum of individual fatty acid relative percentages. Temperature of roasting were indicated in the legend table above. The acronym "nr" means "value not recorded"

Table S3

Analysis of total phenols (free+bound), antioxidant activity and individual phenols in: 1) raw hazelnut with skin (R_S), and 2) toasted hazelnut without skin (T_WS) from different origin: (3A) Turkish grown in Turkey (Tombul), (3B) Italian hazelnut grown in Italy (TGT-TG) and (3C) Italian hazelnut grown in Chile (TGT-Chile).

A

**PHENOLS and A.A. ACTIVITY**	**Turkish Hazelnut grown up in Turkey. Tombul variety**							
	**Raw with Skin (R_S)**			**Toasted Without Skin (T_WS)**				**Text page**
**Total phenol content (GAE mg/g)**	**TOMBUL Av**	SD	CV	**TOMBUL**	**Av.**	SD	CV	
	**mg/g**			**mg/g**				
	**20.05** (7)	0.04	0.00	4.27 (8) 4.38 (9)	**4.32** (8), (9)	0.08	0.02	#10 #11
	13,7 (13)	0,5						
**Antioxidant activity (DPPH assay, I%)**	**93.60%**	0.3	0.00	27.5% (8) 26.6% (9)	**27.05 %**	0.64	0.02	#10 #11
**PHENOLIC ACIDS**	**TOMBUL Av**	SD	CV	**TOMBUL**	**Av.**	SD	CV	
	**µg/g**			**µg/g**				
Gallic acid	2.39 (7)	0.11	0.04	12.51 (8), 12.36 (9)	12.43 (8),(9)	0.1	0.01	#10 #11
	6.79 (11)	1.72	0.25	0.63 (12)	0.63 (12)	0.24	0.38	#10 #11
	127 (13)	5		nr	nr	nr	nr	#10 #11
Protocatechuic acid	5.31 (7),	0.74	0.14	4.38 (8), 5.26 (9),	4.82 (8), (9)	0.62	0.13	#10 #11
	0.545 (11)	0.23	0.42	0.79 (12)	0.79	0.07	0.09	#10

					(12)			#11
Caffeic acid	1.99 (7)	0.02	0.01	1.82 (8), 2.21 (9),	2.01 (8), (9)	0.27	0.14	#10 #11
	0.08 (11)	0.01	0.12	0.10 (12)	0.10 (12)	0.09	0.9	#10 #11
	81 (13)	2		nr	nr	nr	nr	#10 #11
Salicylic acid	0.20 (11)	0.05	0.25	0.72 (12)		0.05	0.07	#10 #11
Siringic acid	1.42 (11)	0.10	0.07	2.84 (12)		2.88	1.01	#10 #11
Vanillic acid	2.39 (11)	0.25	0.10	3. 96 (12)		3.59	0.91	#10 #11
4-Hydroxybenzoic acid	0.21 (11)	0.06	0.28	0.70 (12)		0.51	0.73	#10 #11
Ferulic acid	1.05 (11)	0.12	0.11	1.24 (12)		0.83	0.67	#10 #11
	105 (13)	5		nr		nr	nr	#10 #11
o-Coumaric acid	1.86 (11)	0.26	0.14	1.94 (12)		1.61	0.83	#10 #11
p-Coumaric acid	208 (13)	15		nr		nr	nr	#10 #11
Sinapic Acid	3.13 (11)	0.89	0.28	1.37 (12)		0.39	0.28	#10 #11
	93 (13)	5						

B

**PHENOLS and A.A. ACTIVITY**	**Italian hazelnut grown up in Italy. TGT-TG varieties**										
	**Raw with Skin (R_S)**					**Toasted without Skin (T_WS)**					**Text page**
**Total phenol content (GAE mg/g)**	**TGT Piedmont**	**TG Campania**	**Av.**	SD	CV	**TGT Piedmont**	**TG Campania**	**Av.**	SD	CV	
	**mg/g**					**mg/g**					
	17.56 (7*)	10.21 (7*)	**13.88**	5.19	0.37	3.9 (8*), 2.88 (9*)	4.09 (8*) 2.25 (9*)	**3.28**	0.15	0.05	#10 #11
Antioxidant activity (DPPH assay, I %)	88.05 % (7*)	84.9 % (7*)	**87 %**	2.74	0.03	30.2% (8*), 30.0 % (9*)	21.0% (8*) 21.2 % (9*)	**25.6 %**	6.36	0.25	#10 #11
**PHENOLIC ACIDS**	**TGT_ Piedmont**	**TG_ Campania**	**Av.**	SD	CV	**TGT_ Piedmont**	**TG_ Campania**	**Av.**	SD	CV	
	**µg/g**					**µg/g**					

Gallic acid	2.50 (7*)	1.85 (7*)	**<u>2.18</u>**	0.46	0.21	6.81 (8*), 7.05 (9*)	12.29 (8*); 6.51 (9*)	**<u>8,17</u>**	3.1	0.4	#10 #11
Protocatechuic acid	5.31 (7*)	5.78 (7*)	**<u>5.54</u>**	0.33	0.06	5.59 (8*), 6.63 (9*);	3.55 (8*); 8.11 (9*)	**<u>5.97</u>**	1.92	0.32	#10 #11
Caffeic acid	2.13 (7*)	1.63 (7*)	**<u>1.88</u>**	0.35	0.19	2.38 (8*), 2.45 (9*);	1.94 (8*), 2.05 (9*)	**<u>2.21</u>**	0.25	0.11	#10 #11
pHydroxybenzoic acid	1.73 (7*)	2.73 (7*)	**<u>2.23</u>**	0.7	0.32	2.36 (8*)2.55 (9*)	1.87 (8*) 2.34 (9*)	**<u>2.28</u>**	0.29	0.13	#10 #11

C

PHENOLS and A.A. ACTIVITY	Italian Hazelnut grown up in Chile TGT variety							
	Raw with Skin (R_S)			Toasted Without Skin (T_WS)				Text pages
**Total phenol content (GAE mg/g)**	**TGT-Chile AV**	**SD**	**CV**	**TGT-Chile**	**Av.**	**SD**	**CV**	
	**mg/g**			**mg/g**				
	13.67 (7+)	0.85	0.06	4.05 (8+) 4.66 (9+)	4.35	0.43	0.09	#10 #11
**Antioxidant activity (DPPH assay, I%)**	**90.4%**	0.4	0.00	38.2 (8+) 39.0(9+)	**38.6**	0.56	0.01	#10 #11
**PHENOLIC ACIDS**	**TGT-Chile**	**SD**	**CV**	**TGT-Chile**	**Av.**	**SD**	**CV**	
	**µg/g**			**µg/g**				
Gallic acid	2.98 (7+)	0.11	0.04	8.57 (8+) 10.83 (9+	9.7	1.60	0.16	#10 #11
Protocatechuic acid	6.59 (7+)	0.76	0.11	5.82 (8+) 4.71 (9+)	5.26	0.78	0.15	#10 #11
Caffeic acid	2.51 (7+)	0.06	0.02	2.46 (8+) 2.41 (9+)	2.43	0.03	0.01	#10 #11
pHydroxybenzoic acid	4.01 (7+)	0.36	0.08	2.01 (8+) 2.1 (9+)	2.05	0.06	0.03	#10 #11

Table S3A,B,C Legend

Publications	State of production	Hazelnut type	Hazelnut processing	Skin
7) Locatelli et al. 2015 [5]	7) Turkey	Tombul	**(R_S)**	**Yes**
	7*) Italy	TGT_TG		
	7+) Chile	TGT_Chile		
8) Locatelli et al. 2015 [5]	8) Turkey	Tombul	**(T_WS) at 160 °C**	**No**
	8*) Italy	TGT_TG		
	8+) Chile	TGT_Chile		
9) Locatelli et al. 2015 [5]	9) Turkey	Tombul	**(T_WS) at 180 °C**	**No**
	9*) Italy	TGT_TG		

	9+) Chile	TGT_Chile		
11) Pelvan et al.2018 [7]	Turkey	Tombul	**(R_S)**	**Yes**
12) Pelvan et al.2018 [7]	Turkey	Tombul	**(T_WS)**	**No**
13) Shahidi et al. 2007 [8]	Turkey	Tombul	**(R_S)**	**Yes**

Concentration values of **total phenols (free+bound), antioxidant activity (%) and individual phenols** of raw hazelnut with skin (R_S) and toasted hazelnut without skin (T_WS) cultured Turkey, in Italy or Chile were obtained from two publications (Locatelli et al.2015 and Pelvan et al.2015) as indicated in the table above. Where necessary, average, DS and CV were calculated separately. Data were expressed as mg/g or µg/g of hazelnut. Temperature of roasting were also indicated in the legend table above. The acronym "nr" means "value not recorded"

In the study by Locatelli et al 2015, the units of measurement in the table for phenolic acid values were incorrectly written in table in mg/g instead of µg/g, which was instead correctly written in the text of the article (confirmation of the units of measurement was received from the author herself). Whereas total phenols (GAE) are correctly written in mg/g.

Table S4

Concentration analysis of mineral salts in the Turkish Tombul raw with skin (R_S) hazelnut and toasted without skin (T_WS) hazelnut.

**MINERAL SALTS**	**Turkish Hazelnut grown in Turkey**								
	**Raw with Skin (R_S)**				**Toasted without Skin (T_WS)**				**Text page**
**ESSENTIAL MACRO-ELEMENTS**	**TOMBUL**	**Av.**	SD	CV	**TOMBUL**	**Av.**	SD	CV	
	**mg/100g**				**mg/100g**				
Ca	193.40 (1), 181.83 (6), 164.00 (10), 143.70(14)	170.73	21.70	0.13	135.40 (15) 148.30 (16)	141.85	9.12	0.06	#11
P	335.70 (1), 300.66	331.79	29.37	0.09	nr	nr	nr	nr	#11

	(6), 359.00 (10)								
Mg	176.5 (1) 146.83 (6) 192 (10), 127.60 (14)	160.73	28.97	0.18	119.2 (15), 152.4 (16)	135.8	23.48	0.17	#11
K	761.00 (1), 599.60 (6) 666.00 (10)	675.53	81.12	0.12	6.04 (16)	6.04	0.11	0.01	#11
Na	3.13 (1), 2.73 (6), nd (10)	2.93	0.28	0.10	661.00 (16)	661.00	3.2	0.00	#11
**Total essential macroelements (mg/100g)**		**1009.93** (Ca, Mg, K, Na)	161.4	0.16		**944.69** (Ca, Mg, K, Na)	32.6	0.03	#11
**ESSENTIAL MICRO-ELEMENTS**	**mg/100g**	**Av.**	SD	CV	**mg/100g**	**Av.**	SD	CV	**Text page**
Fe	4.97(1), 3.93 (6), 3.80 (10), 2.60 (14)	3.83	0.97	0.25	2.23 (15), 3.43 (16)	2.83	0.84	0.3	#11
Zn	1.94 (1), 2.62 (6), 2.56 (10), 1.60 (14)	2.18	0.49	0.23	1.41 (15) 3.44 (16)	2.42	1.44	0.6	#11
**Total essential microelements (mg/100g)**		**6.01**	1.46	0.24		**5.25**	2.29	0.43	#11
**NOT ESSENTIAL MICRO-ELEMENTS**	**mg/100g**	**Av.**	SD	CV	**mg/100g**	**Av.**	SD	CV	**Text page**
Cu	1.6 (1), 1.69 (6), 1.65 (10), 1.37 (14)	1.58	0.14	0.009	1.31 (15), 0.84 (16)	1.07	0.33	0.31	#11
Mn	3.29 (1), 6.13 (6), 6.45 (10), 5.34 (14)	5.30	1.42	0.27	5.01 (15), 1.38 (16)	3.20	2.57	0.80	#11

Cr	0.01 (1), 0.006 (14)	0.01	0.00	0.35	0.007(15), 0.031 (16)	0.02	0.02	0.89	#11
Se	0.060 (1), nd (10), 0.002 (14)	0.03	0.04	1.33	0.0010 (15), 0.07 (16)	0.04	0.05	1.37	#11
Mo	0.04 (6)	0.04	/	/	0.04 (16)	0.04	/	/	#11
Si	0.99 (14)	0.99	/	/	0.94 (15) 18.71 (16)	9.83	12.57	1.28	#11
Ni	1.25 (1) 0.15 (14)	0.70	0.78	0.11	0.27 (16)	0.27	/	/	#11
Sn	0.004 (14)	0.00	/	/	0.004 (15)	0.00	/	/	#11
B	2.78 (6), 1.6 (14)	2.19	0.83	0.38	1.29 (15), 5.05 (16)	3.17	2.66	0.84	#11
Co	0.22 (1), 0.027 (14)	0.12	0.14	1.11	0.03 (15), 0.01 (16)	0.02	0.01	0.65	#11
**Total not-essential microelements**		**10.96**	3.36	0.30		**17.65**	18.20	1.03	#11
**TOXIC MICRO - ELEMENTS**	**mg/100g**	**Av.**	SD	CV	**mg/100g**	**Av.**	SD	CV	
Al	5.02 (1) 0.44 (14)	2.73	3.24	1.19	0.29 (15),	0.29	0.0	1.41	#11
As	0.005(14)	0.005	/	/	0.006 (15)	0.006	0.00	0.00	#11
Cd	0.010 (1) 0.006 (14)	0.008	0.00	0.35	0.001 (15)	0.001	0.00	0.00	#11
Pb	0.03 (1) 0.009 (14)	0.02	0.01	0.71	0.008 (15), 0.044 (16)	0.026	0.03	0.96	#11
Ag	0.010 (1)	0.01	0.0	0.0	nr	/	/	/	#11
**Total toxic microelements**		**2.77**	3.25	2.25		**0.32**	0.03	0.08	#11

Table S4 Legend

Publications	State of production	Hazelnut type	Hazelnut processing	Skin
1) Alasalvar et al. 2003 [1]	Turkey	Tombul	**(R_S)**	**Yes**
6) Ozenc et al. 2014 [4]	Turkey	Tombul	**(R_S)**	**Yes**
10) Schlormann et al 2015 [6]	Turkey	Tombul	**(R_S)**	**Yes**
14) Kafaoglu et al. 2014 [9]	Turkey	Tombul	**(R_S)**	**Yes**
15) Kafaoglu et al 2014 [9]	Turkey	Tombul	**(T_WS)**	**No**
16) Tosic et al. 2015 [10]	Turkey	Tombul	**(T_WS)**	**No**

Concentration values of mineral salts of raw hazelnut with skin (R_S) and toasted hazelnut without skin (T_WS) cultured in Turkey were obtained from five publications and were expressed as mg/100g of product as indicated. Average, DS and CV were calculated. In this case, authors didn't indicate the Temperature of roasting. The acronym 'nd' means 'unavailable value'. The acronym "nr" means "value not recorded".

Table S5

Analyses of vitamins in the Tombul and Viba Sweet Turkish hazelnut by comparing raw with skin (R_S) with toasted without skin (T_WS) hazelnuts.

**VITAMINS**	**Turkish Hazelnut grown in Turkey. Tombul- Viba sweet types**								
	**Raw with Skin (R_S)**				**Toasted Without Skin (T_WS)**				**Text Page**
	**TOMBUL-VIBA SWEET** (mg/100g)	**Average** (mg/100g)	SD	CV	**TOMBUL-VIBA SWEET** (mg/100g)	**Average** (mg/100g)	SD	CV	
vitamin A precursor (beta – carothens)	n.d. (1), 0.03 (2)	0.03	0.00	0.00	0.02 (3) 0.02 (4)	0.02	0.00	0.00	#12 #13
vitamin B1 (thiamine)	0.42 (1), 0.31 (2)	0.37	0.08	0.21	0.28 (3) 0.24 (4)	0.26	0.03	0.12	#12 #13
vitamin B2 (riboflavin)	0.10 (1), 0.37 (2)	0.24	0.19	0.81	0.38 (3), 0.39 (4)	0.39	0.00	0.01	#12 #13
vitamin B3 (niacin)	1.94 (1)	1.94	0.15	0.07	nr	/	/	/	#12 #13
vitamin B5 (pantothenic acid)	1.12 (1)	1.12	0.07	0.06	nr	/	/	/	#12 #13
vitamin B6 (pyridoxine)	0.63 (1), 0.47 (2)	0.55	0.11	0.21	0.55 (3), 0.58 (4)	0.57	0.02	0.04	#12 #13
vitamin B7 (biotin)	0.08 (1)	0.08	0.01	0.12	nr	/	/	/	#12 #13
vitamin B9 (folate, folic acid)	0.12 (1)	0.12	0.01	0.08	nr	/	/	/	#12 #13
vitamin B12 (Cobalamin)	n.d. (1)	/	/	/	Nr	/	/	/	#12 #13
vitamin C (ascorbic acid)	5.54 (1)	5.54	0.23	0.04	nr	/	/	/	#12 #13

vitamin E_ active form (α-tocopherols)	24.00 (1), 40.60 (2),19.10 (10)	27.9	11.3	0.41	34.0 (3), 32.6(4), 12.73(10*), 11.82(10°)	22.79	12.16	0.53	#12 #13
vitamin E (ß-tocopherol)	1.15 (10)	1.15	0.07	0.06	0.75 (10*) 0.57 (10°)	0.66	0.13	0.19	#12 #13
vitamin E (γ-tocopherol)	9.5 (2) 4.64 (10)	7.07	3.44	0.49	5.10 (3), 6.60 (4), 4.57 (10*) 4.41 (10°)	5.17	1.0	0.2	#12 #13
vitamin E (δ-tocopherol)	n.d. (10)	/	/	/	n.d. (10*)	/	/	/	n.d.
vitamin E (tocotrienols)	n.d. (10)	n.d.	/	/	n.d. (10*)	n.d.	/	/	n.d.
**Total vitamins (mg/100g)**	**46.11 (A,B,C,E)**		15.66	0.34	**vit. C not analyzed**		/	/	#12 #13
	**40.57 (A,B,E)**		15.43	0.38	**29.86 (A,B,E)**		13.34	0.45	
**Total B group vitamins (mg/100g)**	**4.21 (B1-B6)**		0.60	0.14	**1.21 (B1-B6)**		0.05	0.04	#12 #13
	**4.41 (B1-B12)**		0.62	0.14	**B7, B9, B12 not analyzed**		/	/	#12 #13
**Total antioxidant vitamins (mg/100g)**	**41.70 (A,C,E)**		15.04	0.36	**vit. C not analyzed**		/	/	#12 #13
	**36.16 (A,E)**		14.81	0.41	**28.63 (A,E)**		13.29	0.46	#12 #13

Table S5 Legend

**Publications**	**State of production**	**Hazelnut type**	**Hazelnut processing**	**Skin**
1) Alasalvar et al. 2003 [1]	Turkey	Tombul	**(R_S)**	**Yes**
2) Stuets et al. 2017 [2]	Turkey	Viba Sweets	**(R_S)**	**Yes**
3) Stuets et al. 2017 [2]	Turkey	Viba Sweets	**(T_WS)_ 140 °C**	**No**
4) Stuets et al. 2017 [2]	Turkey	Viba Sweets	**(T_WS) 170 °C**	**No**
10) Schlormann et al. 2015 [6]	Turkey	Tombul	**(R_S)**	**Yes**
10*) Schlormann et al. 2015 [6]	Turkey	Tombul	**(T_WS) 140 °C**	**No**
10°) Schlormann et al. 2015 [6]	Turkey	Tombul	**(T_WS) 180 °C**	**No**

Data were obtained from three different publications as indicated in the table above. *Vitamin c*oncentrations were expressed as mg/100g of product. In the R_S hazelnut were analyzed A, B1, B2, B3, B5, B6, B7, B9, B12, C and E vitamins, whereas in the T_WS hazelnut A, B1, B2, B3, B6 and E vitamins ((α,ß,γ,δ tocopherols and tocotrienols). The acronym 'nd' means 'unavailable value'. The acronym "nr" means "value not recorded".

Table S6

Analysis of flavonoid quantity contained in 1) raw hazelnut with skin (R_S) and 2) toasted hazelnut without skin (T_WS) from different origin: (A) Turkish grown in Turkey (Tombul), (B) Italian hazelnut grown in Italy (TGT Piedmont-TG Campania) and (C) Italian hazelnut grown in Chile (TGT-Chile).

A

**FLAVONOIDS**	**Turkish Hazelnut grown in Turkey Tombul type**								
	**Raw_with Skin (R_S)**				**Toasted_Without Skin (T_WS)**				**Text page**
	**Tombul**	**Av**	SD	CV	**Tombul**	**Av**	SD	CV	
	**µg/g**				**µg/g**				
Catechin	7.32 (7)	7.32 (7)	0.23	0.03	9.53(8) 11.61 (9)	10.57	1.47	0.14	#12 #13
Epicatechin	3.02 (7)	3.02 (7)	0.29	0.10	2.62 (8) 2.68 (9)	2.65	0.04	0.02	#12 #13
Kaempferol	3.58 (7)	3.58 (7)	0.41	0.11	3.03(8) 3.50 (9)	3.26	0.33	0.10	#12 #13
Myricetin	7.05 (7)	7.05 (7)	0.41	0.06	6.00(8) 6.82(9)	6.41	0.58	0.09	#12 #13
Quercitin	7.50 (7)	7.50 (7)	0.03	0.00	5.84(8) 6.47 (9)	6.15	0.45	0.07	#12 #13
**Total flavonoids (µg/g)**		**28.47**	1.37	0.30		**29.05**	2.87	0.41	#12 #13

B

FLAVONOIDS	Italian Hazelnut grown in Italy TGT_TG										
	**Raw with Skin (R_S)**					**Toasted Without Skin (T_WS)**					**Text page**
	**TGT_ Piedmont**	**TG_ Campania**	**Av**	SD	CV	**TGT_ Piedmont**	**TG_ Campania**	**Av**	SD	CV	
	**µg/g**					**µg/g**					
Catechin	5.59 (7) 5.39 (7)	9.55 (7)	6.84	2.35	0.34	20.95 (8) 11.08 (8) 16.42 (9) 11.29 (9)	8.45 (8) 14.95 (9)	13.86	4.51	0.33	#12 #13
Epicatechin	2.2 (7) 4.89 (7)	2.13 (7)	2.94	1.34	0.46	2.87(8) 2.83(8) 2.81 (9) 2.89 (9)	2.96 (8) 2.55 (9)	2.82	0.14	0.05	#12 #13
Kaempferol	2.92 (7) 4.42 (7)	2.65 (7)	3.33	0.95	0.29	3.88 (8) 4.02(8) 3.75 (9) 4.01 (9)	3.14 (8) 3.33 (9)	3.69	0.37	0.1	#12 #13
Myricetin	5.52 (7) 8.16 (7)	5.04 (7)	6.24	1.68	0.27	7.12 (8) 7.11(8) 6.95 (9) 7.42 (9)	5.98 (8) 6.31 (9)	6.82	0.55	0.08	#12 #13
Quercitin	5.40 (7) 7.93 (7)	5.84 (7)	6.39	1.35	0.21	8.1(8), 7.44(8) 7.7 (9) 7.55(9)	6.99 (8) 7.80 (9)	7.60	0.37	0.05	#12 #13
**Total flavonoids (µg/g)**	26.20	25.21	**25.74**	7.67	0.30	36.53	31.21	**34.78**	5.94	0.61	#12 #13

C

FLAVONOIDS	Italian Hazelnut grown in Chile TGT-Chile								
	Raw with Skin (R_S)				Toasted Without Skin (T_WS)				Text page
	TGT_Chile	Av	SD	CV	TGT_Chile	Av	SD	C.V.	
	µg/g				µg/g				
Catechin	7.55 (7)	7.55	2.10	0.28	12.57 (8) 12.01 (9)	12.29	0.40	0.03	#12 #13
Epicatechin	3.58 (7)	3.58	0.20	0.06	3.07 (8) 3.25 (9)	3.16	0.13	0.04	#12 #13
Kaempferol	4.34 (7)	4.34	0.41	0.09	3.73 (8) 3.82 (9)	3.77	0.06	0.02	#12 #13
Myricetin	8.10 (7)	8.10	0.10	0.01	6.99 (8) 7.13 (9)	7.06	0.10	0.01	#12 #13
Quercitin	7.99 (7)	7.99	0.34	0.04	7.40 (8) 7.43 (9)	7.41	0.02	0.00	#12 #13
**Total flavonoids (µg/g)**		**31.56**	3.15	0.09		**33.7**	0.71	0.02	#12 #13

Table S6 legend

Publications	State of production	Hazelnut type	Hazelnut processing	Skin
7) Locatelli et al. 2015 [5]	Turkey or Italy or Chile	Tombul form Turkey / TGT and TG from Italy /TGT from Chile	**(R_S)**	**Yes**
8) Locatelli et al. 2015 [5]	Turkey or Italy or Chile	Tombul from Turkey / TGT and TG from Italy /TGT from Chile	**(T_WS) at 160 °C**	**No**
9) Locatelli et al. 2015 [5]	Turkey or Italy or Chile	Tombul from Turkey/ TGT and TG from Italy /TGT from Chile	**(T_WS) at 180 °C**	**No**

Flavonoid quantifications were expressed as **µg/g** of product as described in Locatelli M. et al.2015. Where necessary, concentration average together with SD and CV were calculated separately. Total flavonoids were calculated by the sum of individual compounds. Temperature of roasting were indicated in the legend table above.

In the study by Locatelli et al 2015, the units of measurement in the table of flavonoid values were erroneously written in mg/g instead of µg/g, as was correctly written in the text of the article (confirmation of the units of measurement was received from the author herself).

Table S7

Skin analysis of Giresun hazelnut cultivated in Turkey, Tonda Gentile delle Langhe in Chile (TGL_Chile), Tonda Gentile delle Langhe in Italy (TG_Campania) and Tombul in Turkey was added to Table B.

A

**PHENOLS / FLAVAN-3-OLS**	**Giresun grow in Turkey**	**Tonda Gentile delle Langhe grow in Chile**	**Tonda Gentile delle Langhe grow in Italy**	**Tonda di Giffoni grow in Italy**	**Text page**
Catechin (mg/100 g)	153,10 (17)	99.90 (17^)	193,10 (17*)	190,00 (17*)	#14
Epicatechin (mg/100 g)	30,20 (17)	11.90 (17^)	75,90 (17*)	22,30 (17*)	#14
Epicatechin/3-O gallate (mg/100 g)	4,90 (17)	0.70 (17^)	10,20 (17*)	3,00 (17*)	#14
Procyanidin dimers (mg/100 g)	278,70 (17)	133.20 (17^)	341,60 (17*)	277,50 (17*)	#14
Procyanidin dimer gallates (mg/100 g)	18,50 (17)	3.40 (17^)	21,30 (17*)	7,70 (17*)	#14
Procyanidin trimers (mg/100 g)	26,50 (17)	4.00 (17^)	14,70 (17*)	16,00 (17*)	#14
Prodelphinidin dimers (mg/100 g)	48,70 (17)	25.20 (17^)	112,60 (17*)	72,50 (17*)	#14
Prodelphinidin trimers (mg/100 g)	4,30 (17)	nr	5,90 (17*)	4,60 (17*)	nr

B

**PHENOLIC ACIDS**	**Tombul grow in Turkey**	**Giresun grow in Turkey**	**Tonda Gentile delle Langhe grow in Chile**	**Tonda Gentile delle Langhe grow in Italy**	**Tonda di Giffoni grow in Italy**	**Text page**
Gallic acid (mg/100g)	38,70 (12) 801,30 (13)	1,90 (17)	3.00 (17^)	3,90 (17*)	4,00 (17*)	#14
Protocatechuic acid (mg/100g)	0,10 (13)	nr	2.40 (17^)	1,80 (17*)	2,20 (17*)	#14
p-coumaric (mg/100g)	23,10	nr	nr	nr	nr	nr

	(12)					
o-coumaric (mg/100g)	4,12 (13)	nr	nr	nr	nr	nr
caffeic (mg/100g)	0,11 (13) Trace (12)	nr	nr	nr	nr	nr
sinapic (mg/100g)	12,40 (12) 2,37 (13)	nr	nr	nr	nr	nr
Salicylic acid (mg/100g)	0.01 (13)	nr	nr	nr	nr	nr
Syringic acid (mg/100g)	0,98 (13)	nr	nr	nr	nr	nr
Vanillic acid (mg/100g)	3,31 (13)	nr	nr	nr	nr	nr
4-Hydroxybenzoic acid (mg/100g)	0.01 (13)	nr	nr	nr	nr	nr
ferulic (mg/100g)	12,40 (12) 1,01(13)	nr	nr	nr	nr	nr

C

**FLAVONOLS**	**Giresun grow in Turkey**	**Tonda Gentile delle Langhe grow in Chile**	**Tonda Gentile delle Langhe grow in Italy**	**Tonda di Giffoni grow in Italy**	**Text page**
Quercetin (mg/100g)	1,80 (17)	1.60 (17^)	2,70 (17*)	1,50 (17*)	#14
Quercetin 3-O rhamnoside (mg/100g)	8,10 (17)	4.70 (17^)	9,40 (17*)	7,20 (17*)	#14
Myricetin (mg/100g)	0,30 (17)	0.20 (17^)	0,50 (17*)	0,20 (17*)	#14
Myricetin rhamnoside (mg/100g)	1,00 (17)	0.50 (17^)	1,70 (17*)	1,00 (17*)	#14
Kaempferol rhamnoside (mg/100g)	0,50 (17)	0.40 (17^)	0,40 (17*)	0,40 (17*)	#14

D

**DIHYDROCHALCONE**	**Giresun grow in Turkey**	**Tonda Gentile delle Langhe grow in Chile**	**Tonda Gentile delle Langhe grow in Italy**	**Tonda di Giffoni grow in Italy**	**Text page**
Phloretin 2-O glucoside (mg/100g)	10,30 (17)	8.80 (17^)	17,40 (17*)	19,00 (17*)	#14

E

	**Giresun grow in Turkey**	**Tonda Gentile delle Langhe**	**Tonda Gentile delle Langhe**	**Tonda di Giffoni grow in Italy**	**Text page**

		grow in Chile	grow in Italy		
TOTAL POLYPHENOL (g/100g)	8,60 (17)	4.10 (17^)	11,10 (17*)	8,70 (17*)	#14

Table S7 A,B,C,D,E Legend

Publications	State of production	Hazelnut type
12) Shahidi et al. 2007 [8]	Turkey	Tombul
13) Pelvan et al. 2018 [7]	Turkey	Tombul
17) Del Rio, D. et al. 2011 [11]	17) Turkey	Giresun
	17*) Italy	TGT_TG
	17^) Chile	TGT

"nr" means "value not recorded"

Table S8

Analysis of hazelnut oil from different origin: Turkish grown in Turkey (Tombul), Italian hazelnut Tonda Giffoni grown in Portugal (TG_Portugal) and in New Zealand (TG_New Zealand)

	Hazelnut Oil							
**Fat acids and tocopherols**	**Tombul grown in Turkey**	**AV**	**DS**	**TG grown in New Zealand**	**TG grown in Portugal**	**AV**	**DS**	**Text page**
Linoleic acid (18:2 ω6) %	8.89 (18)<br>8.85 (19)<br>9.07 (23)	8.94	0.13	12.53 (21)	10.29 (22)	11.41	1.58	#18
Linoleic acid (18:3 ω3) %	0.1 (18)<br>0.12 (19)	0.11	0.01	0.12 (21)	0.10 (22)	0.11	0.01	#18
Oleic acid 18:1 ω9 %	82.72 (18)<br>82.78 (19)<br>85.12 (23)	83.54	1.37	78.97 (21)	80.14 (22)	79.56	0.83	#18
Palmic acid (16:0) %	4.85 (18)<br>4.81 (19)<br>4.95 (23)	4.87	0.07	4.83 (21)	5.14 (22)	4.98	0.22	#18
Stearic acid (18:0) %	2.73 (18)<br>2.69 (19)<br>0.59 (23)	2.00	1.22	1.95 (21)	2.46 (22)	2.21	0.36	#18
Beta-Sitosterol (mg/100 g)	105.48 (18)<br>132.05 (19)<br>133 (20)	123.51	15.62	145.30 (21)	162.89 (22)	154.10	12.01	#18

Campesterol (mg/100 g)	7.15 (18)<br>9.92 (19)<br>6.28 (20)	7.78	1.90	8.00 (21)	9.72 (22)	8.86	1.22	#18
Stigmasterol (mg/100 g)	0.89 (18)<br>1.61 (19)<br>1.24 (20)	1.25	0.36	1.50 (21)	1.79 (22)	1.65	0.21	#18
Total sterol content (mg/100 g)	113.52 (18)<br>164.92 (19)<br>153 (20)	143.81	26.90	nr	nr			nr
Total Tocols	51.31 (19)<br>46.9 (20)	49.11	3.12	nr	nr			nr
Total vitamin E = Total Tocopherol (mg/100 g)	43.45 (18)<br>36.1 (20)	39.78	5.20	44.72 (21)	nr	44.72		nr
α-Tocopherols (mg/100 g)	38.23 (18)<br>40.4 (19)<br>34.5 (20)	37.71	2.98	40.39 (21)	nr	40.39		nr
α-Tocotrienol (mg/100 g)	0.2 (17)<br>0.27 (18)	0.24	0.05	nr	nr			nr
β -Tocotrienol (mg/100 g)	0.09 (19)<br>0.08 (20)	0.09	0.01	nr	nr			nr
β-Tocopherols (mg/100 g)	1.15 (18)<br>1.53 (19)<br>1.42 (20)	1.37	0.20	1.30 (21)	nr	1.30		nr
γ-Tocotrienol (mg/100 g)	0.23 (19)<br>0.31 (20)	0.27	0.06	nr	nr			nr
γ -Tocopherols (mg/100g)	3.89 (18)<br>8.33 (19)<br>9.93 (20)	7.38	3.13	2.78 (21)	nr	2.78		nr
δ -Tocopherols (mg/100g)	0.18 (18)<br>0.53 (19)<br>0.45 (20)	0.39	0.18	0.25 (21)	nr	0.25		nr
Total SFA (Saturated Fatty Acid) (g/100g)	7.85 (18)<br>7.79 (19)<br>5.69 (23)	7.11	1.23	nr	7.81 (22)	7.81		nr
Total MUFA (Monouns. fatty acid) (g/100g)	38.16 (18)<br>83.24 (19)	60.70	31.88	nr	81.75 (22)	81.75		nr
Total PUFA (g/100g)	8.99 (18)<br>8.97 (19)	8.98	0.01	12.70 (21)	10.39 (22)	11.55	1.63	#18

Table S8 legend

Publications	State of production	Hazelnut type
18) Alasalvar C., Shahidi, F., Ohshima, at al 2003 [12]	Turkey	Tombul
19) Alasalvar C., Amaral, J.S, Shahidi, F. 2006 [13]	Turkey	Tombul
20) Alasalvar C. at al 2009 [14]	Turkey	Tombul
21) Savage et al. 1997 [15]	New Zealand	Tonda di Giffoni

22) Amaral J.S et al 2006 [16]	Portugal	Tonda di Giffoni
23) Kanbur et al. 2013 [17]	Turkey	Tombul

The acronym "nr" means "value not recorded"

References

1. Alasalvar, C.; Shahidi, F.; Liyanapathirana, C.M.; Ohshima, T. Turkish Tombul Hazelnut (*Corylus avellana* L.). 1. Compositional Characteristics. *J. Agric. Food Chem.* **2003**, *51*, 3790–3796.

2. Stuetz, W.; Schlörmann, W.; Glei, M. B-vitamins, carotenoids and α-/γ-tocopherol in raw and roasted nuts. *Food Chem.* **2017**, *221*, 222–227.

3. Erdemir, U.S.; Gucer, S. Bioaccessibility of Copper in Turkish Hazelnuts (Corylus avellana L.) by Chemical Fractionation and In Vitro Methods. *Biol. Trace Elem. Res.* **2015**, *167*, 146–154.

4. Özenç, N.; Bender Özenç, D. Nut traits and nutritional composition of hazelnut (*Corylus avellana* L.) as influenced by zinc fertilization. *J. Sci. Food Agric.* **2015**, *95*, 1956–1962.

5. Locatelli, M.; Coïsson, J.D.; Travaglia, F.; Bordiga, M.; Arlorio, M. Impact of Roasting on Identification of Hazelnut (Corylus avellana L.) Origin: A Chemometric Approach. *J. Agric. Food Chem.* **2015**, *63*, 7294–7303.

6. Schlörmann, W.; Birringer, M.; Böhm, V.; Löber, K.; Jahreis, G.; Lorkowski, S.; Müller, A.K.; Schöne, F.; Glei, M. Influence of roasting conditions on health-related compounds in different nuts. *Food Chem.* **2015**, *180*, 77–85.

7. Pelvan, E.; Olgun, E.Ö.; Karadağ, A.; Alasalvar, C. Phenolic profiles and antioxidant activity of Turkish Tombul hazelnut samples (natural, roasted, and roasted hazelnut skin). *Food Chem.* **2018**, *244*, 102–108.

8. Shahidi, F.; Alasalvar, C.; Liyana-Pathirana, C.M. Antioxidant Phytochemicals in Hazelnut Kernel (*Corylus avellana* L.) and Hazelnut Byproducts. *J. Agric. Food Chem.* **2007**, *55*, 1212–1220.

9. Kafaoğlu, B.; Fisher, A.; Hill, S.; Kara, D. Chemometric evaluation of trace metal concentrations in some nuts and seeds. *Food Addit. Contam. Part A* **2014**, *31*, 1529–1538.

10. Tošić, S.B.; Mitić, S.S.; Velimirović, D.S.; Stojanović, G.S.; Pavlović, A.N.; Pecev-Marinković, E.T. Elemental composition of edible nuts: fast optimization and validation procedure of an ICP-OES method. *J. Sci. Food Agric.* **2015**, *95*, 2271–2278.

11. Del Rio, D.; Calani, L.; Dall'Asta, M.; Brighenti, F. Polyphenolic Composition of Hazelnut Skin. *J. Agric. Food Chem.* **2011**, *59*, 9935–9941.

12. Alasalvar, C.; Shahidi, F.; Ohshima, T.; Wanasundara, U.; Yurttas, H.C.; Liyanapathirana, C.M.; Rodrigues, F.B. Turkish Tombul Hazelnut (*Corylus avellana* L.). 2. Lipid Characteristics and Oxidative Stability. *J. Agric. Food Chem.* **2003**, *51*, 3797–3805.

13. Alasalvar, C.; Amaral, J.S.; Shahidi, F. Functional Lipid Characteristics of Turkish Tombul Hazelnut (*Corylus avellana* L.). *J. Agric. Food Chem.* **2006**, *54*, 10177–10183.

14. Alasalvar, C.; Amaral, J.S.; Satır, G.; Shahidi, F. Lipid characteristics and essential minerals of native Turkish hazelnut varieties (Corylus avellana L.). *Food Chem.* **2009**,

113, 919–925.

15. Savage, G.P.; McNeil, D.L.; Dutta, P.C. Lipid composition and oxidative stability of oils in hazelnuts (*Corylus avellana* L.) grown in New Zealand. *J. Am. Oil Chem. Soc.* **1997**, *74*, 755–759.

16. Amaral, J.S.; Casal, S.; Seabra, R.M.; Oliveira, B.P.P. Effects of Roasting on Hazelnut Lipids. *J. Agric. Food Chem.* **2006**, *54*, 1315–1321.

17. Kanbur G.; Arslan D.; Ozcan M. M. Some compositional and physical characteristics of some Turkish hazelnut (Corylus avellana L.) variety fruits and their corresponding oils. *Int. Foor Res. J.* **2013**, *20*, 2161–2165.
